# Facility-based surveillance for influenza and respiratory syncytial virus in rural Zambia

**DOI:** 10.1186/s12879-021-06677-5

**Published:** 2021-09-21

**Authors:** Gideon Loevinsohn, Mutinta Hamahuwa, Pamela Sinywimaanzi, Katherine Z. J. Fenstermacher, Kathryn Shaw-Saliba, Andrew Pekosz, Mwaka Monze, Richard E. Rothman, Edgar Simulundu, Philip E. Thuma, Catherine G. Sutcliffe

**Affiliations:** 1 Department of Epidemiology, Johns Hopkins University Bloomberg School of Public Health, 615 N. Wolfe Street, Room E6535, Baltimore, MD 21205 USA; 2 Johns Hopkins University School of Medicine, Baltimore, MD USA; 3 Macha Research Trust, Macha, Choma, Zambia; 4 Department of Emergency Medicine, Johns Hopkins University School of Medicine, Baltimore, MD USA; 5 Department of Microbiology and Immunology, Johns Hopkins University Bloomberg School of Public Health, Baltimore, MD USA; 6 Virology Laboratory, University Teaching Hospital, Lusaka, Zambia

**Keywords:** Influenza, RSV, Southern Africa, Rural, Risk factors, Severity

## Abstract

**Background:**

While southern Africa experiences among the highest mortality rates from respiratory infections, the burden of influenza and respiratory syncytial virus (RSV) in rural areas is poorly understood.

**Methods:**

We implemented facility-based surveillance in Macha, Zambia. Outpatients and inpatients presenting with influenza-like illness (ILI) underwent testing for influenza A, influenza B, and RSV and were prospectively followed for 3 to 5 weeks to assess clinical course. Log-binomial models assessed correlates of infection and clinical severity.

**Results:**

Between December 2018 and December 2019, 17% of all outpatients presented with ILI and 16% of inpatients were admitted with an acute respiratory complaint. Influenza viruses and RSV were detected in 17% and 11% of outpatient participants with ILI, and 23% and 16% of inpatient participants with ILI, respectively. Influenza (July–September) and RSV (January-April) prevalence peaks were temporally distinct. RSV (relative risk [RR]: 1.78; 95% confidence interval [CI] 1.51–2.11), but not influenza, infection was associated with severe disease among patients with ILI. Underweight patients with ILI were more likely to be infected with influenza A (prevalence ratio [PR]: 1.72; 95% CI 1.04–2.87) and to have severe influenza A infections (RR: 2.49; 95% CI 1.57–3.93).

**Conclusions:**

Populations in rural Zambia bear a sizeable burden of viral respiratory infections and severe disease. The epidemiology of infections in this rural area differs from that reported from urban areas in Zambia.

**Supplementary Information:**

The online version contains supplementary material available at 10.1186/s12879-021-06677-5.

## Background

Influenza viruses and respiratory syncytial virus (RSV) are leading causes of global respiratory morbidity and mortality [1, 2]. In southern Africa their burden is becoming increasingly evident, with each nascent surveillance effort uncovering a considerable prevalence of disease [3, 4]. Available evidence suggests that southern Africa experiences among the highest worldwide influenza- and RSV-related mortality rates [2, 5]. In South Africa, for example, influenza-related mortality among elderly adults was found to be over three-fold higher than in the United States [6]. Prevalent malnutrition, HIV infection, and tuberculosis increase the severity of viral illness and may contribute to mortality [7–9]. However, much remains unknown about viral epidemiology and risk factors for severe disease. The overwhelming majority of surveillance and research efforts in southern Africa have been based in urban centers. Information from rural areas is particularly scant, yet this is where the majority of the region’s population lives [10]. Rural environments present distinct risk factors for transmission and pathogenesis given lower population density, reduced access-to-care, and higher prevalence of undernutrition and extreme poverty [11]. Urban–rural differences in epidemiology and severity have been previously described in sub-Saharan Africa for many infectious diseases including tuberculosis [12], HIV [13], and malaria [14].

In Zambia, published studies on influenza and RSV have been limited to the urban capital of Lusaka and government-led influenza surveillance has been confined to Lusaka and the city of Ndola [4, 15–17]. There are no disease estimates from rural areas and few estimates overall among adult populations [15, 18]. In the absence of context-specific disease estimates for rural areas, clinicians and public health practitioners have little insight into respiratory disease etiology and risk factors for severe illness, likely contributing to antibiotic overuse and misallocation of scarce clinical resources.

In December 2018, facility-based surveillance for influenza and RSV was established in rural Zambia to evaluate their role in causing respiratory illness and begin to situate rural Zambia in the landscape of regional and global virus transmission. The objective of this analysis was to describe the burden of influenza and RSV disease during the first year of surveillance and explore predictors of severe disease.

## Methods

### Study site and population

Facility-based surveillance was established at Macha Hospital in Southern Province, Zambia as part of the Johns Hopkins Center for Excellence in Influenza Research and Surveillance (JHCEIRS) with the primary goal of understanding the burden and epidemiology of influenza virus in this area. Macha Hospital is a 208-bed, district-level hospital that serves a catchment population of approximately 150,000, predominantly subsistence farmers. Southern Zambia historically experiences three seasons: a single rainy season from November to April, a cool dry season from May to August, and a warm dry season from September to November [19]. Malaria prevalence in the area has steadily declined and was < 1% in recent years [20]. Provincial vaccination coverage for *Streptococcus pneumoniae* and *Hemophilus influenzae* type b among children 12–23 months of age is estimated at 91% for the full complement of vaccine doses [21]. Influenza vaccines and antiviral medications are unavailable in the study area.

### Surveillance procedures

Beginning on December 10, 2018, all outpatients presenting for care to the outpatient department (OPD) and patients newly admitted to the adult and pediatric wards with respiratory symptoms were screened for influenza-like illness (ILI). ILI was defined based on the Centers for Disease Control and Prevention (CDC) definition as measured (≥ 38 °C) or reported fever with either cough or sore throat, either with clinical onset and/or worsening within 7 days of hospital presentation [22]. Reported fever was added to the CDC case definition to increase sensitivity and to account for prior antipyretic use. All inpatients and an age-stratified weekly sample of outpatients with ILI were eligible for enrollment (Additional file 1). Enrollment days and times in the OPD were varied to capture the breadth of the outpatient population.

At enrollment, a nasopharyngeal specimen was collected using a flocked swab, placed in universal transport media (*Cepheid Inc.*, Sunnyvale, CA), and stored at 4 ℃. Participants were administered a questionnaire by trained study staff detailing sociodemographic characteristics, symptoms, medical history, and potential exposures. Food insecurity was assessed using the Household Hunger Scale, a three-question scale validated for cross-cultural use [23]. HIV serostatus, assessed through routine hospital screening, was recorded. Weight, height, respiratory rate, and peripheral capillary oxygen saturation (%SpO_2_; measured using a handheld pulse oximeter [*CMI Health Inc.*, Alpharetta, GA]) were captured at enrollment. Treatment administered and patient disposition were obtained from the medical record.

Study participants were followed three to five weeks after enrollment to ascertain clinical course. Information on vital status, symptoms, and hospital admissions were obtained via self-report and by reviewing the medical record. For participants who could not be reached in person, the information was collected by telephonic interview.

### Laboratory procedures

Nasopharyngeal specimens were transported to the Clinical Research Laboratory of the Macha Research Trust within one hour of collection. The specimens were tested for influenza A/B viruses and RSV using the Cepheid Xpert Xpress Flu/RSV assay (*Cepheid Inc.,* Sunnyvale, CA) on the day of collection. The Xpert Xpress Flu/RSV assay has demonstrated a sensitivity/specificity of 98.6%/99.3% and 97.9%/99.4% for detection of influenza A and B viruses, respectively, and 98.1%/99.4% for RSV when compared with gold-standard laboratory-based RT-PCR assays [24].

### Temperature and precipitation

Temperature and precipitation were captured once per hour using an outdoor HOBO Micro Station weather sensor (*Onset Computer Corporation*, Bourne, MA).

### Statistical analyses

This analysis reports on the results of surveillance carried out from December 10, 2018 to December 9, 2019. Characteristics of the study population were summarized at enrollment. Participants without a test result for influenza virus or RSV were excluded from the analysis (n = 2; due to death and withdrawal, respectively). Monthly prevalence of influenza virus and RSV infection among all outpatients was calculated using direct standardization based on the age distribution and age-specific ILI prevalence in the outpatient population, and the age-specific prevalence of influenza virus and RSV among those with ILI enrolled in the study. As all inpatients were not systematically screened for ILI, the monthly prevalence of influenza virus and RSV infection was only estimated among inpatients with ILI, and was calculated directly as the proportion of participants with influenza virus and RSV infection. For both patient populations, trends in respiratory illness (ILI for outpatients and acute respiratory infection for inpatients), influenza virus, and RSV prevalence over time were graphically summarized with locally weighted scatterplot smoothing (LOWESS) techniques.

Differences in presenting characteristics between groups defined by viral testing results were assessed among both outpatient and inpatient participants with univariable and age-adjusted log-binomial models. Where the models failed to converge, Poisson regression with robust variance estimation was employed. Separate models were fit for the outcomes of influenza A virus, influenza B virus, and RSV infection respectively. Sociodemographic characteristics, medical history, and presenting symptoms were evaluated as predictors of viral infection. Moderate or severe hunger was defined as a value of two or greater on the Household Hunger Scale [23]. For children 18 years or younger, underweight was defined as a body mass index (BMI) more than two standard deviations below the age- and sex-specific mean using the WHO’s Child Growth Standards [25]. For adults, underweight was defined as a BMI less than 18.5. Tachypnea was defined based on age-specific cutoffs [26]. To evaluate factors associated with severe clinical illness among all participants testing positive for influenza virus or RSV infection, age-adjusted log-binomial models were fit with the outcome of severe clinical illness (vs non-severe clinical illness) separately for influenza A virus, influenza B virus, and RSV. Severe clinical illness was defined as a composite outcome encompassing at least one of: death while under follow-up, respiratory illness requiring hospital admission at enrollment or during follow-up, or peripheral oxygen saturation (SpO_2_) ≤ 92% at enrollment [27, 28]. Participants with missing information for one or more of these outcomes (n = 114 for death and hospital admission during follow-up, n = 134 for SpO_2_) who were not already designated as severe based on available data were excluded from the analysis (n = 168, 25% of participants).

Statistical analyses were performed using STATA version 14 (*Statacorp,* College Station, TX*).*

## Results

### Influenza-like illness

**Fig. 1:**
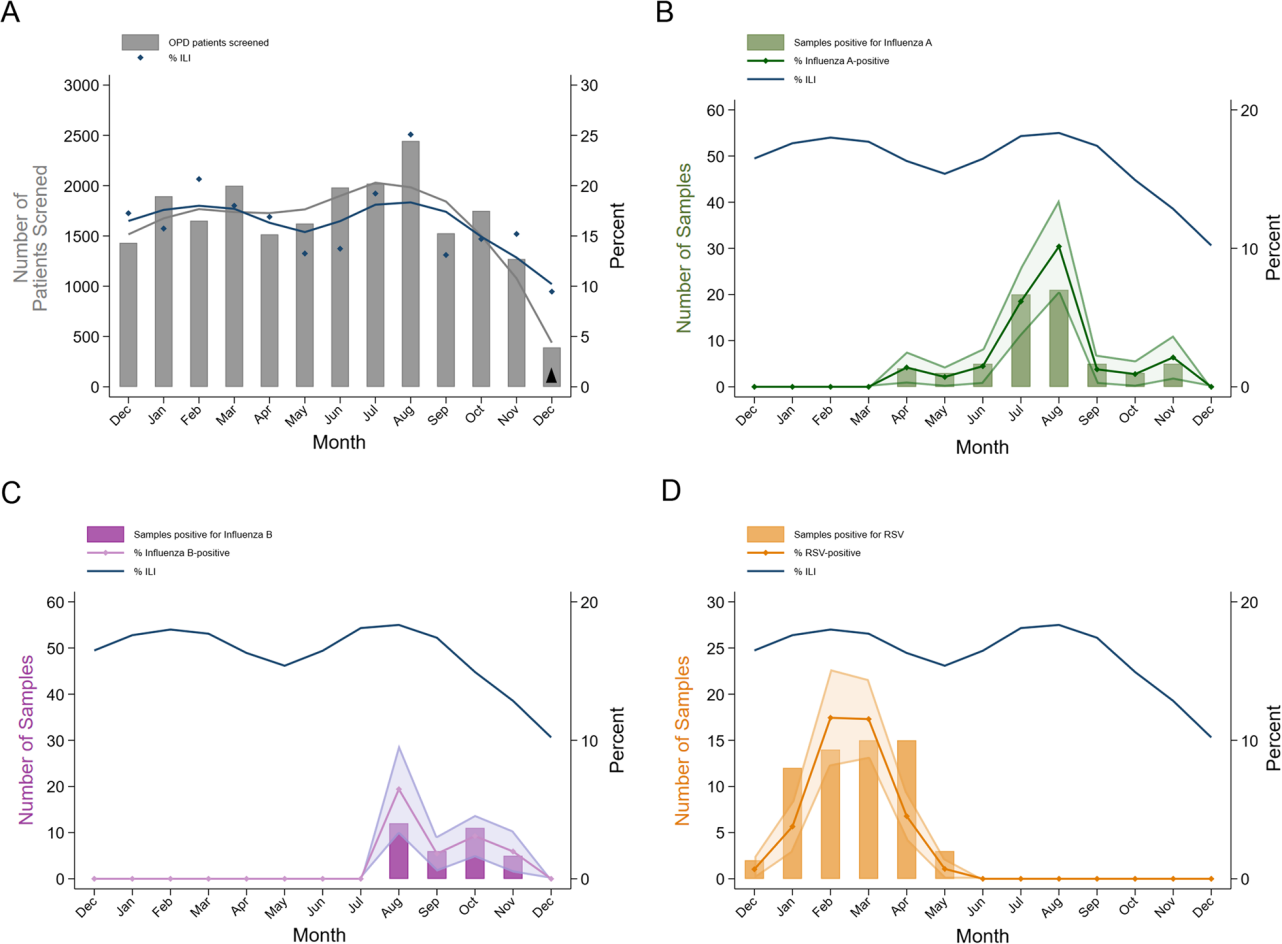
Trends in (**A**) ILI, **B** influenza A virus, **C** influenza B virus, and **D** RSV prevalence among outpatients. ILI: influenza-like illness; OPD: outpatient department; RSV: respiratory syncytial virus. Black up pointing triangle: Partial month of surveillance. **A** OPD attendance and proportion of OPD patients with ILI at Macha Hospital. Gray and blue lines are LOWESS curves for number of patients screened and proportion with ILI, respectively. **B**–**D** Viral positivity over the study period for influenza A, influenza B, and RSV, respectively. Bar graph represents the number of positive tests among OPD study participants. Line represents estimated prevalence among all OPD patients with 95% confidence interval. Blue line represents estimated monthly ILI prevalence among all OPD patients (LOWESS)

Between December 10, 2018 and December 9, 2019, 3,677 of 21,492 (17%) outpatients presenting to the Macha Hospital OPD met criteria for ILI. The highest prevalence was observed among patients under 1 year of age and 1–4 years (0–11 months: 712/1615 [44%]; 1–4 years: 1264/2885 [44%]; 5–15 years: 536/2586 [21%]; 16–50 years: 898/11,187 [8%]; 51+ years: 240/3098 [8%]). ILI was common throughout the year, with peaks in prevalence observed from February-April and July–August (Fig. 1A). In age-stratified analysis, the first ILI peak (February-April) was observed only in children under 15 years, while the second peak (July–August) was observed across age-groups (Additional file 3).

**Fig. 2:**
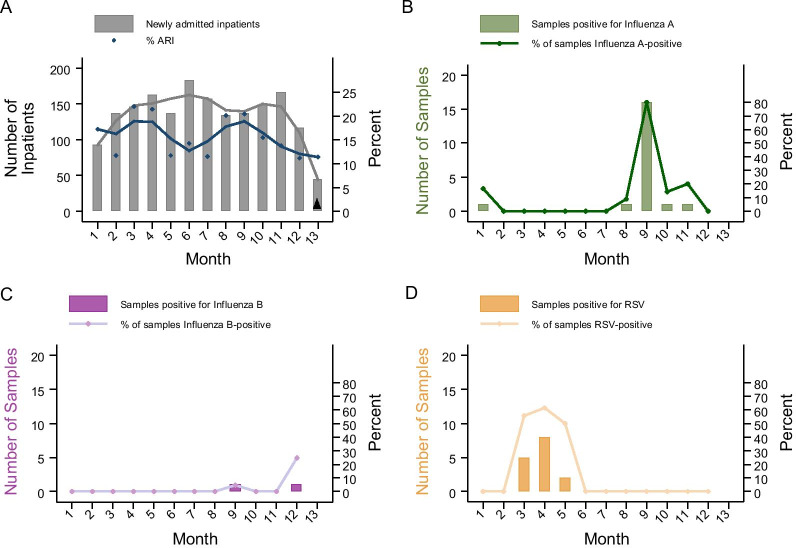
Trends in (**A**) ARI among all newly admitted patients, and **B** influenza A virus, **C** influenza B virus, and **D** RSV prevalence among hospitalized patients with ILI. ARI: acute respiratory illness; ILI: influenza-like illness; RSV: respiratory syncytial virus. Black up pointing triangle: Partial month of surveillance. **A** Newly admitted patients and proportion of all inpatients with ARI at Macha Hospital. Gray and blue lines are LOWESS curves for the number of newly admitted patients and proportion with ARI, respectively. **B**–**D** Viral positivity over the study period for influenza A, influenza B, and RSV, respectively, among inpatients with ILI. Bar graph and line represent the number and proportion of positive tests among inpatient study participants with ILI, respectively

During the surveillance period, 279 of 1781 patients (16%) were admitted with an acute respiratory complaint. Of those screened for enrollment, 166 met criteria for ILI and inclusion in the study. Similar trends over time in the prevalence of acute respiratory infections were found among hospitalized patients as in the outpatient population (Fig. 2).

### Influenza viruses and RSV

Among outpatients with ILI, 650 eligible patients (17% of outpatients with ILI) were approached for enrollment: 74 (11%) declined participation and 576 (89%) were enrolled in the study (Table 1). In addition, 166 eligible inpatients were approached: 70 declined participation (42%) and 95 (58%) were enrolled. The most common reason for declining was the absence of family decision-makers (42% of refusals). There were no significant differences in age (p = 0.33) or sex (p = 0.58) between those declining participation and those enrolled. The age distribution of participants closely mirrored the distribution of respiratory illness in the patient populations (Additional files 4 and 5).

**Table 1:** Characteristics of the study population at enrollment in Macha, Zambia

	Outpatients	Inpatients	Overall
	n = 576	n = 95	n = 671
Age in years, median (IQR)	3.2 (0.8–19.0)	2.9 (0.8–29.0)	3.2 (0.8–19.0)
0–5 months	61 (11%)	11 (12%)	72 (11%)
6–11 months	100 (17%)	15 (16%)	115 (17%)
1–4 years	181 (31%)	31 (33%)	212 (32%)
5–15 years	77 (13%)	9 (9%)	86 (13%)
16–50 years	91 (16%)	18 (19%)	109 (16%)
51+ years	66 (11%)	11 (12%)	77 (11%)
Female, n (%)	316 (55%)	44 (46%)	360 (54%)
Educational attainment among adults^a^, n (%)			
No education	11 (7%)	1 (4%)	12 (7%)
Primary	101 (68%)	20 (77%)	121 (69%)
Secondary	34 (23%)	2 (8%)	36 (21%)
Post-secondary	3 (2%)	3 (12%)	6 (3%)
Travel time (hours) to hospital, median (IQR)	1.0 (0.8–2.0)	2.0 (1.0–3.0)	1.0 (0.8–2.0)
Number of individuals in household, median (IQR)	6 (5–9)	7 (5–11)	7 (5–9)
Number of individuals sharing sleeping space, median (IQR)	3 (2–3)	3 (2–3)	3 (2–3)
Presence of animals in residence compound, n (%)	528 (92%)	84 (88%)	612 (91%)
Food insecurity, n (%)	14 (2%)	6 (6%)	20 (3%)
Underweight, n (%)	47 (8%)	21 (22%)	68 (10%)
HIV-infected, n (%)	19 (3%)	10 (11%)	29 (4%)
History of tuberculosis, n (%)	16 (3%)	6 (6%)	22 (3%)
Number of outpatient medical encounters in past year, median (IQR)^b^	1 (0–3)	1 (0–2)	1 (0–3)

^a^Limited to participants 18 years or older ^b^Excluding the enrollment visit

Among outpatient participants, 66 (11%) were found to be infected with influenza A virus, 34 (6%) with influenza B virus, and 61 (11%) with RSV. The estimated prevalence among all outpatients presenting for care, regardless of symptoms, was 2.06% for influenza A virus (95% confidence interval [CI] 1.59%–2.54%), 1.09% for influenza B virus (95% CI 0.73%–1.45%), and 1.80% for RSV (95% CI 1.38%–2.20%). Among all outpatients presenting with ILI, the estimated prevalence was 12.07% (95% CI 9.28%–14.85%) for influenza A virus, 6.39% (95% CI 4.29%–8.49%) for influenza B virus, and 10.51% (95% CI 8.04%–12.98%) for RSV.

Among all outpatients, distinct temporal trends were observed for both influenza virus and RSV infections during the surveillance period (Fig. 1B–D). RSV infections were detected from December 2018 to May 2019. While this coincides with the historical rainy season, the study coincided with a period of regional drought (Additional file 6). In contrast, influenza viruses were detected during the cold-dry and warm-dry seasons. Influenza A virus was detected from April to November 2019 with the highest prevalence, an estimated 10.13% of all outpatients (95% CI 6.71%–13.56%), in August. Influenza B virus was detected from August to November 2019, with the highest prevalence, an estimated 6.47% of all outpatients (95% CI 3.22%–9.72%), also in August. The two peaks in ILI prevalence coincided with the peaks in RSV and influenza virus prevalence (Fig. 1B–D).

Among inpatient participants with ILI, 20 (21%) were found to be infected with influenza A virus, 2 (2%) with influenza B virus, and 15 (16%) with RSV. Similar temporal trends were observed as for the outpatient population (Fig. 2) with RSV peaking early in the year followed by a peak in influenza virus prevalence later in the dry seasons.

### Symptomatology of influenza virus and RSV infections among patients with ILI

Participants (outpatient and inpatient) with ILI presented to Macha Hospital a median of 3 days after symptom onset (IQR 3–4; Additional file 2). There were no significant differences in the duration of symptoms by viral test result. Among children younger than 5 years of age, measured fever (≥ 38 ℃) was significantly associated with influenza A virus infection (Prevalence ratio [PR]: 2.29; 95% CI 1.34–3.91) but not influenza B virus or RSV infection. Those presenting with headache were significantly more likely to be infected with influenza B virus (PR: 9.78; 95% CI 1.06–90.18) and those presenting with diarrhea were significantly less likely to be infected with RSV (PR: 0.50; 95% CI 0.27–0.92). The presence of hypoxemia (SpO_2_ ≤ 92%) was significantly associated with RSV infection (PR: 3.10; 95% CI 1.75–5.49). Among participants older than 5 years, measured fever was significantly associated with influenza A virus (PR: 2.28; 95% CI 1.19–4.34) and influenza B virus (PR: 7.62; 95% CI 3.01–19.26) infection, but not RSV. In addition, the presence of tachypnea was significantly associated with influenza A virus infection (PR: 2.90; 95% CI 1.32–6.39).

### Risk factors for influenza virus and RSV infection among patients with ILI

Inpatient participants were significantly more likely to be infected with influenza A virus than outpatient participants (age-adjusted [adj] PR 1.86; 95% CI 1.19–2.89; Table 2). There were no significant differences in influenza B virus or RSV prevalence by inpatient status. Influenza A and influenza B viruses were detected across age groups (Table 2, Additional file 3). In contrast, RSV infections were concentrated among children younger than 5 years of age.

**Table 2:** Risk factors for influenza and RSV infection among participants with influenza-like illness in Macha, Zambia

	Total n = 671	Influenza A n = 86			Influenza B n = 36				RSV n = 76			
		Prevalence^a^	Univariable prevalence ratio (95% CI)	Age-adjusted prevalence ratio (95% CI)	Prevalence^a^	Univariable prevalence ratio (95% CI)	Age-adjusted prevalence ratio (95% CI)		Prevalence^a^		Univariable prevalence ratio (95% CI)	Age-adjusted prevalence ratio (95% CI))
Sociodemographic characteristics and household exposures												
Age, years												
Per year increase			1.00 (0.99–1.01)	–		0.97 (0.95–1.00)		–			0.94 (0.91–0.97)	–
0–5 months	72	7 (10%)	Ref	–	4 (6%)	Ref		–	14 (19%)		Ref	–
6–11 months	115	6 (5%)	0.54 (0.19–1.53)	–	2 (2%)	0.31 (0.06–1.67)		–	16 (14%)		0.72 (0.37–1.38)	–
1–4	212	37 (17%)	1.80 (0.84–3.85)	–	14 (7%)	1.19 (0.40–3.50)		–	35 (17%)		0.85 (0.49–1.49)	–
5–15	86	17 (20%)	2.03 (0.89–4.63)	–	12 (14%)	2.51 (0.85–7.45)		–	8 (9%)		0.48 (0.21–1.08)	–
16–50	109	10 (9%)	0.94 (0.38–2.37)	–	4 (4%)	0.66 (0.17–2.56)		–	2 (2%)		**0.09 (0.02–0.40)**	–
51+	77	9 (12%)	1.20 (0.47–3.06)	–	0 (0%)	N/A		–	1 (1%)		**0.07 (0.01–0.50)**	–
Patient type (n,%)												
Outpatient	576	66 (11%)	Ref	Ref	34 (6%)	Ref		Ref	61 (11%)		Ref	Ref
Inpatient	95	20 (21%)	**1.84 (1.17–2.88)**	**1.86 (1.19–2.89)**	2 (2%)	0.36 (0.09–1.46)		0.38 (0.09–1.56)	15 (16%)		1.49 (0.89–2.51)	1.54 (0.93–2.56)
Sex (n,%)												
Male	311	44 (14%)	Ref	Ref	11 (4%)	Ref		Ref	41 (13%)		Ref	Ref
Female	360	42 (12%)	0.82 (0.56–1.22)	0.81 (0.55–1.20)	11 (4%)	1.96 (0.98–3.93)		1.95 (0.98–3.90)	41 (13%)		0.74 (0.48–1.13)	0.86 (0.56–1.31)
Recent travel outside district (n, %)												
No	636	79 (12%)	Ref	Ref	34 (5%)	Ref		Ref	76 (12%)		Ref	Ref
Yes	34	7 (21%)	1.66 (0.83–3.31)	**2.35 (1.14–4.84)**	2 (6%)	1.10 (0.28–4.39)		1.77 (0.42–7.48)	0 (0%)		N/A	N/A
Symptomatic contact in household (n,%)												
No	328	32 (10%)	Ref	Ref	14 (4%)	Ref		Ref	28 (9%)		Ref	Ref
Yes	337	52 (15%)	**1.58 (1.05–2.39)**	**1.54 (1.02–2.31)**	22 (7%)	1.53 (0.80–2.94)		1.51 (0.80–2.88)	48 (14%)		**1.67 (1.07–2.59)**	**1.57 (1.02–2.43)**
Indoor cooking (n,%)												
No	616	82 (13%)	Ref	Ref	30 (5%)	Ref		Ref	66 (11%)		Ref	Ref
Yes	55	4 (7%)	0.55 (0.21–1.43)	0.52 (0.20–1.36)	6 (11%)	2.24 (0.97–5.15)		1.89 (0.83–4.28)	10 (18%)		1.70 (0.93–3.11)	**2.00 (1.12–3.56)**
Current smoker or smoker in household (n,%)												
No	547	68 (12%)	Ref	Ref	33 (6%)	Ref		Ref	64 (12%)		Ref	Ref
Yes	124	18 (15%)	1.17 (0.72–1.89)	1.19 (0.74–1.91)	3 (2%)	0.40 (0.12–1.29)		0.39 (0.12–1.25)	12 (10%)		0.83 (0.46–1.48)	0.85 (0.48–1.50)
Number of individuals sharing sleeping space												
Per person increase	3 (2–3)	3 (2–3)	0.93 (0.75–1.14)	0.89 (0.71–1.13)	3 (2–3)	1.11 (0.84–1.46)		1.02 (0.74–1.39)	3 (3–3)		1.14 (0.98–1.33)	1.00 (0.81–1.24)
1	29	3 (10%)	Ref	Ref	0 (0%)	Ref		Ref	0 (0%)		Ref	Ref
2–3	512	69 (13%)	1.30 (0.44–3.89)	1.21 (0.39–3.73)	28 (5%)	N/A		N/A	58 (11%)		N/A	N/A
4+	130	14 (11%)	1.04 (0.32–3.39)	1.28 (0.30–5.54)	8 (6%)	N/A		N/A	18 (14%)		N/A	N/A
Food insecurity (n,%)												
No	652	85 (13%)	Ref	Ref	36 (6%)	Ref		Ref	75 (12%)		Ref	Ref
Yes	19	1 (5%)	0.40 (0.06–2.75)	0.51 (0.08–3.45)	0 (0%)	N/A		N/A	1 (5%)		0.46 (0.07–3.12)	0.60 (0.09–3.93)
Recent animal exposure^b^ (n,%)												
No	555	67 (12%)	Ref	Ref	30 (5%)	Ref		Ref	67 (12%)		Ref	Ref
Yes	112	17 (15%)	1.26 (0.77–2.06)	1.16 (0.71–1.92)	6 (5%)	0.99 (0.42–2.32)		0.90 (0.38–2.12)	9 (8%)		0.67 (0.34–1.30)	0.98 (0.51–1.92)
Medical history												
Underweight (n,%)												
No	601	71 (12%)	Ref	Ref	33 (5%)	Ref		Ref	69 (11%)		Ref	Ref
Yes	68	14 (21%)	**1.74 (1.04–2.92)**	**1.72 (1.04–2.87)**	3 (4%)	0.80 (0.25–2.55)		0.99 (0.32–3.10)	6 (9%)		0.77 (0.35–1.70)	0.96 (0.44–2.08)
HIV-infected (n,%)												
No	639	83 (13%)	Ref	Ref	36 (6%)	Ref		Ref	76 (12%)		Ref	Ref
Yes	29	3 (10%)	0.80 (0.27–2.37)	0.97 (0.31–3.01)	0 (0%)	N/A		N/A	0 (0%)		N/A	N/A
History of tuberculosis (n,%)												
No	649	85 (13%)	Ref	Ref	36 (6%)	Ref		Ref	76 (12%)		Ref	Ref
Yes	22	1 (5%)	0.35 (0.05–2.38)	0.39 (0.06–2.80)	0 (0%)	N/A		N/A	0 (0%)		N/A	N/A
History of chronic cardiovascular or pulmonary disease (n,%)												
No	637	83 (13%)	Ref	Ref	36 (6%)	Ref		Ref	76 (12%)		Ref	Ref
Yes	34	3 (9%)	0.68 (0.23–2.03)	0.73 (0.22–2.43)	0 (0%)	N/A		N/A	0 (0%)		N/A	N/A
Anemia (n,%)												
No	650	84 (13%)	Ref	Ref	36 (6%)	Ref		Ref	73 (11%)		Ref	Ref
Yes	21	2 (10%)	0.74 (0.19–2.80)	0.77 (0.20–2.92)	0 (0%)	N/A		N/A	3 (14%)		1.27 (0.44–3.71)	2.21 (0.82–5.92)

^a^Percentages represent row totals (i.e. the total number with a given attribute within each infection group divided by the total number with a given attribute)

^b^Recent animal contact is defined as handling or feeding in the 5 days preceding hospital presentation; includes contact with whole animal carcass but not with processed meat

CI, Confidence interval; N/A, Not available; REF, Reference group; Bold = p < 0.05 from log-binomial regression

With regard to household exposures, the presence of an individual with respiratory symptoms in the household was associated with both influenza A virus (adjPR: 1.54; 95% CI 1.02–2.31; Table 2) and RSV infection (adjPR: 1.57; 95% CI 1.02–2.43). Indoor cooking was associated with RSV infection (adjPR: 2.00; 95% CI 1.12–3.56) but not influenza A or B virus infection. There were few differences in the prevalence of viral infection between those with and without medical comorbidities. However, participants who were underweight were more likely to be infected with influenza A than participants who were not underweight (adjPR: 1.72; 95% CI 1.04–2.87).

### Treatment, outcomes, and clinical severity among patients with ILI

At the time of presentation, participants with influenza A virus infection were more likely to be diagnosed with sepsis than participants without influenza A (adjPR: 3.50; 95% CI 1.42–8.62), participants with influenza B were more likely to be diagnosed with a respiratory tract infection than participants without influenza B (adjPR: 1.20; 95% CI 1.03–1.39), and participants with RSV were more likely to be diagnosed with pneumonia than participants without RSV (adjPR: 3.21; 95% CI 1.59–6.48; Table 3). Of note, 75% of all participants presenting with ILI, including 87% of participants with either influenza virus or RSV infection were prescribed antibiotics. The likelihood of antibiotic prescription was higher for participants with influenza A virus (adjPR: 1.12; 95% CI 1.02–1.25) or RSV (adjPR: 1.20; 95% CI 1.10–1.31) than those uninfected.

**Table 3:** Clinical care and outcomes among participants with influenza-like illness in Macha, Zambia

	Influenza A				Influenza B				RSV			
	Negative (n = 585)	Positive (n = 86)	Univariable PR/RR (95% CI)	Age-adjusted PR/RR (95% CI)	Negative (n = 635)	Positive (n = 36)	Univariable PR/RR (95% CI)	Age-adjusted PR/RR (95% CI)	Negative (n = 595)	Positive (n = 76)	Univariable PR/RR (95% CI)	Age-adjusted PR/RR (95% CI)
Pneumonia diagnosis at presentation (n,%)	33 (6%)	7 (8%)	1.44 (0.66–3.16)	1.57 (0.71–3.45)	40 (6%)	0 (0%)	N/A	N/A	29 (5%)	11 (14%)	**2.97 (1.55–5.70)**	**3.21 (1.59–6.48)**
RTI diagnosis at presentation (n,%)	412 (70%)	65 (76%)	1.07 (0.94–1.22)	1.05 (0.92–3.45)	446 (70%)	31 (86%)	**1.23 (1.07–1.41)**	**1.20 (1.03–1.39)**	417 (70%)	60 (79%)	1.13 (0.99–1.28)	1.11 (0.98–1.26)
Sepsis diagnosis at presentation (n,%)	14 (2%)	7 (8%)	**3.40 (1.41–8.19)**	**3.50 (1.42–8.62)**	20 (3%)	1 (3%)	0.88 (0.12–6.39)	0.73 (0.10–5.34)	18 (3%)	3 (4%)	1.30 (0.39–4.33)	1.06 (0.32–3.56)
Received antibiotics (n,%)	427 (73%)	73 (85%)	**1.16 (1.05–1.29)**	**1.12 (1.02–1.25)**	470 (74%)	30 (83%)	1.13 (0.97–1.31)	1.07 (0.92–1.25)	431 (72%)	69 (91%)	**1.25 (1.15–1.37)**	**1.20 (1.10–1.31)**
Severe clinical illness (n,%)^a^	133 (32%)	32 (38%)	1.18 (0.95–1.46)	1.18 (0.97–1.43)	160 (34%)	5 (14%)	0.42 (0.21–0.82)	0.56 (0.29–1.11)	132 (29%)	33 (65%)	**1.82 (1.53–2.18)**	**1.78 (1.51–2.11)**
*Inpatient admission (n,%)*^*b*^	85 (17%)	23 (27%)	**1.47 (1.13–1.91)**	**1.42 (1.12–1.79)**	106 (19%)	2 (6%)	0.34 (0.13–0.86)	0.58 (0.23–1.50)	91 (17%)	17 (27%)	**1.45 (1.09–1.94)**	**1.53 (1.20–1.95)**
*Oxygen saturation* ≤ *92% (n,%)*^***c***^	68 (15%)	13 (16%)	1.05 (0.61–1.80)	1.01 (0.58–1.75)	77 (15%)	4 (11%)	0.75 (0.29–1.92)	0.71 (0.27–1.83)	63 (13%)	18 (38%)	**2.91 (1.89–4.48)**	**2.62 (1.68–4.06)**
*Died (n,%)*^***d***^	10 (2%)	0 (0%)	N/A	N/A	10 (2%)	0 (0%)	N/A	N/A	10 (2%)	0 (0%)	N/A	N/A

^a^Among those for whom the occurrence of severe disease could be ascertained (n = 168 excluded). Severe disease defined as any of death during follow-up, admission at enrollment or during follow-up, or SpO^2^ ≤ 92% at enrollment

^b^Includes inpatient admission at enrollment or while under follow-up (n = 114 excluded due to missing data during follow-up)

^c^Measured at enrollment (n = 134 excluded as SpO_2_ measurement was implemented in February 2019)

^d^During follow-up (n = 114 excluded due to missing data during follow-up)

CI, Confidence interval; N/A, Not available; PR, Prevalence ratio; RR, Risk ratio; RTI, respiratory tract illness; Bold = p < 0.05 from log-binomial regression

Overall, clinical severity was assessed in 503 participants (75% of all participants) and 33% (165/503) experienced a severe clinical illness. Ten participants died during follow-up, none of whom were infected with influenza or RSV. Participants with RSV were more likely to have a severe clinical illness than those without RSV (age-adjusted risk ratio [adjRR]: 1.78; 95% CI 1.51–2.11; Table 3), with an increased risk of both hypoxemia (adjPR: 2.62; 95% CI 1.68–4.06) and hospital admission (adjRR: 1.53; 95% CI 1.20–1.95). Overall, no differences were observed in the likelihood of severe clinical illness by influenza virus testing result. Among participants with influenza A virus, being underweight was associated with increased risk of severe illness (adjRR: 2.49; 95% CI 1.57–3.93; Table 4) due to heightened risk of both hospitalization (adjRR: 2.16; 95% CI 1.03–4.52) and hypoxemia (adjPR: 2.96; 95% CI 1.19–7.33). We observed no relationship between HIV infection or prior tuberculosis infection and clinical severity. All participants reporting a diagnosis of HIV also reported receiving combination antiretroviral therapy.

**Table 4:** Correlates of clinical severity among participants with influenza A, influenza B, and respiratory syncytial virus infection in Macha Zambia

	Influenza A			Influenza B			RSV		
	Severe (n = 32)	Univariable RR for severity (95% CI)	Age-adjusted RR for severity (95% CI)	Severe (n = 5)	Univariable RR for severity (95% CI)	Age-adjusted RR for severity (95% CI)	Severe (n = 33)	Univariable RR for severity (95% CI)	Age-adjusted RR for severity (95% CI)
Age, years									
Per year increase		1.00 (0.98–1.01)	–		1.00 (0.91–1.09)	–		0.98 (0.93–1.04)	–
0–5 months	3 (43%)	Ref	–	0 (0%)	Ref^a^	–	8 (80%)	Ref	–
6–11 months	2 (33%)	0.78 (0.19–3.21)	–	0 (0%)		–	9 (82%)	1.02 (0.67–1.55)	–
1–4	17 (49%)	1.13 (0.45–2.85)	–	2 (17%)		–	15 (68%)	0.85 (0.56–1.30)	–
5–15	4 (29%)	0.67 (0.20–2.19)	–	2 (17%)	1.42 (0.23–8.70)	–	0 (0%)	N/A	–
16–50	2 (22%)	0.52 (0.12–2.30)	–	1 (25%)	2.12 (0.25–18.05)	–	0 (0%)	N/A	–
51+	4 (50%)	1.17 (0.39–3.51)	–	0	N/A	–	1 (100%)	N/A	–
Gender									
Male	19 (46%)	Ref	Ref	0 (0%)	Ref	Ref	17 (63%)	Ref	Ref
Female	13 (34%)	0.74 (0.43–1.28)	0.67 (0.39–1.16)	5 (23%)	N/A	N/A	16 (70%)	1.10 (0.74–1.64)	1.00 (0.72–1.40)
Current smoker or smoker in the household (n,%)									
No	27 (43%)	Ref	Ref	3 (10%)	Ref	Ref	28 (70%)	Ref	Ref
Yes	5 (31%)	0.73 (0.33–1.59)	0.72 (0.33–1.58)	2 (100%)	N/A	N/A	5 (50%)	0.71 (0.37–1.37)	0.96 (0.60–1.55)
Underweight (n,%)									
No	21 (32%)	Ref	Ref	5 (17%)	Ref	Ref	29 (63%)	Ref	Ref
Yes	10 (77%)	**2.38 (1.50–3.78)**	**2.49 (1.57–3.93)**	0 (0%)	N/A	N/A	3 (100%)	N/A	N/A
HIV-infected (n,%)									
No	30 (39%)	Ref	Ref	5 (15%)	Ref	Ref	33 (66%)	Ref	Ref
Yes	2 (67%)	1.69 (0.72–3.94)	1.82 (0.55–6.05)	0 (0%)	N/A	N/A	0 (0%)	N/A	N/A
History of tuberculosis (n,%)									
No	31 (40%)	Ref	Ref	5 (15%)	Ref	Ref	33 (66%)	Ref	Ref
Yes	1 (100%)	N/A	N/A	0 (0%)	N/A	N/A	0 (0%)	N/A	N/A
Anemia (n,%)									
No	31 (40%)	Ref	Ref	5 (15%)	Ref	Ref	31 (65%)	Ref	Ref
Yes	1 (50%)	1.24 (0.30–5.10)	1.00 (0.20–4.95)	0 (0%)	N/A	N/A	2 (100%)	N/A	N/A
Objective fever (n,%)									
No	22 (39%)	Ref	Ref	3 (15%)	Ref	Ref	28 (65%)	Ref	Ref
Yes	10 (43%)	1.11 (0.63–1.95)	1.14 (0.66–1.98)	2 (15%)	1.03 (0.20–5.33)	0.95 (0.17–5.30)	5 (71%)	1.10 (0.65–1.84)	1.18 (0.79–1.76)
Diarrhea (n,%)									
No	28 (44%)	Ref	Ref	2 (8%)	Ref	Ref	29 (67%)	Ref	Ref
Yes	4 (27%)	0.60 (0.25–1.45)	0.54 (0.22–1.31)	3 (38%)	4.69 (0.94–23.27)	4.65 (0.88–24.42)	4 (57%)	0.85 (0.43–1.66)	0.69 (0.34–1.40)
Duration of ILI symptoms at presentation, days									
Per day increase		1.05 (0.89–1.23)	1.05 (0.91–1.22)		0.81 (0.48–1.39)	0.76 (0.42–1.36)		1.06 (0.93–1.21)	1.07 (0.93–1.23)
1–2	5 (38%)	Ref	Ref	2 (25%)	Ref	Ref	4 (50%)	Ref	Ref
3–5	24 (42%)	1.09 (0.52–2.32)	1.27 (0.60–2.71)	3 (15%)	0.60 (0.12–2.94)	0.41 (0.07–2.52)	23 (68%)	1.35 (0.65–2.81)	1.31 (0.68–2.54)
6+	3 (33%)	0.87 (0.27–2.74)	0.44 (0.06–3.21)	0 (0%)	N/A	N/A	6 (75%)	1.50 (0.67–3.34)	1.64 (0.77–3.48)

^a^Ages 0–4 years is designated as the reference to enable comparisons across age groups

CI, Confidence interval; ILI, influenza-like illness; N/A, Not available (distribution not significantly different based on Fisher’s exact testing); REF, Reference group; RR, Risk ratio; RTI, respiratory tract infection; Bold = p < 0.05 from log-binomial regression

## Discussion

We report on the findings from 1 year of facility-based surveillance for influenza and RSV in rural Zambia from December 2018 to December 2019. This is among the first efforts to characterize the burden of viral respiratory disease in a rural southern African setting. At our study site more than one in six outpatients presented with ILI and we found a sizeable annual prevalence of both influenza viruses (18%) and RSV (11%) among symptomatic outpatients across age groups. RSV in particular was associated with clinically severe respiratory disease. Overall, 75% of patients presenting with ILI symptoms were prescribed empiric antibiotics, including 87% of those with a viral infection. These findings highlight the burden of influenza viruses and RSV in rural Zambia and the importance of context-specific epidemiologic and etiologic knowledge for clinicians and public health practitioners.

While the study period for the present analysis occurred prior to the start of the COVID-19 pandemic, the research infrastructure and testing platforms described are well-suited, moving forward, to aid in furthering our understanding of influenza viruses and RSV during the current and future outbreaks. Recent reports from the region, including Zambia, highlight the growing concurrent increase in both severe acute respiratory syndrome coronavirus 2 (SARS-CoV-2) infections as well as influenza virus infections [18, 29]. Additionally, the descriptions of clinical course and risk factors for severe disease may be of use to both local clinicians and public health practitioners in treatment and planning functions.

The observed prevalence of influenza virus infection in this study was markedly higher than that reported in published studies from Lusaka where prevalence among outpatients with ILI ranged from 3.7% [30] to 12.6% [4, 15, 16, 30, 31]. These differences may be due to several factors, including differences in the assays used and the age distribution of the study participants. In the study by Mizuta et al. [30], for example, over 43% were children under 1 year, an age group with among the lowest influenza prevalence in our study. However, these differences may also suggest an increased burden of symptomatic respiratory infections in rural areas. Our work complements emerging data from the Zambian Ministry of Health-led surveillance in urban centers suggesting that 2019 may have been a year of particularly intense influenza activity [18].

In this rural setting, we found a distinct but prolonged period of influenza virus activity with cases detected from April to November. These findings contrast both with published estimates of seasonality in Zambia, which suggest a single prevalence peak during the cold, dry season (May–August), [15, 30], and with multi-year data from ongoing surveillance efforts from urban clinical care centers in Lusaka and Ndola that suggest nearly year-round transmission with multiple annual peaks [18]. With regard to RSV, prior studies from Lusaka [15, 17, 32] and other southern African countries [33, 34] report prolonged RSV seasons and multiple annual peaks in RSV incidence, along with temporal coincidence of RSV and influenza virus activity. However, in this study these infections had distinct peaks and were separately responsible for driving a bimodal temporal distribution of respiratory cases in the outpatient population.

Our study period coincided with a period of significant drought in southern Zambia. Given notable associations of respiratory virus transmission with precipitation and humidity [35, 36], this may have impacted the observed temporal patterns of viral prevalence and could explain, at least in part, the differences observed between our study site and northerly urban areas that experienced more rainfall. Troublingly, drought conditions exacerbated existing food insecurity and undernutrition in the region [37]. Our findings of an association between underweight with both influenza A virus infection and more severe clinical influenza illness complement evidence from animal studies and observations among human patients in South Africa [38–40].

This study was among the first to pilot rapid point-of-care viral testing in southern Zambia, an area with little clinical laboratory infrastructure. The Cepheid GeneXpert platform has been broadly adopted in sub-Saharan Africa for use in tuberculosis diagnosis, is increasingly being used for early infant diagnosis and quantification of HIV viral load [41–43], and has most recently been used for rapid diagnosis of SARS-CoV-2 during the current pandemic. Broadening the assay’s scope of use could be key in facilitating sensitive and reliable microbiological testing even in remote, low-resource environments.

This study is not without limitations. First, given the exploratory nature of this study and its relatively small sample size it is difficult to draw causal conclusions on factors associated with clinical disease and severity. Further study in this and other similar settings will be needed to bolster these investigations. Second, as the primary focus of the surveillance platform was influenza virus, a case definition based on ILI was used to identify potential participants. Thus, while our findings may be useful for informing clinical diagnosis of viral infection, we could not detect asymptomatic or pauci-symptomatic infections. In addition, the requirement for fever in the case definition may have led to an underestimation of the burden of symptomatic RSV as a substantial proportion of RSV-infected young children and elderly patients present without fever [44]. Third, 25% of participants were excluded from the assessment of severe clinical illness due to missing data. As retention may be related to poor health outcomes, this may have led to an underestimation of the proportion experiencing severe clinical illness. However, as over half of excluded participants were only missing data on SpO^2^ due to the inclusion of this measure after the study was initiated, this is unlikely to have biased the risk factor analysis. Finally, the present study reports on a single year of viral surveillance. More complete understanding of seasonality and disease patterns will require sustained efforts across multiple years.

In summary, rural southern Zambia bears a large burden of influenza- and RSV- related disease. Rural areas that are home to the majority of national and regional populations have distinct features and risk factors compared to urban centers yet have been consistently under-surveilled to date. Our findings point to important in-country heterogeneity in the prevalence and epidemiology of respiratory infections and highlight the importance of continued, wide-ranging surveillance efforts in capturing the true burden of disease. Such surveillance programs that can provide more granular, context-specific information are key to planning for and responding to existing and emerging disease threats.

## Supplementary Information

**Additional file 1:** Sampling scheme for participants with influenza-like illness recruited from the outpatient department at Macha Hospital

**Additional file 2:** Symptomatology and clinical presentation of influenza virus and RSV infections by age

**Additional file 3:** Age-specific seasonal trends over time in A) influenza-like illness, B) influenza A virus, C) influenza B virus, and D) respiratory syncytial virus prevalence among outpatients in Macha, Zambia, December 2018 to December 2019.

**Additional file 4:** Age-distribution of all outpatients, outpatients with influenza-like illness, and outpatient study participants

**Additional file 5:** Age-distribution of all inpatients, inpatients with acute respiratory illness, and inpatient study participants

**Additional file 6:** Temperature, precipitation, and viral infection in Macha Zambia, December 2018 to December 2019.

## Data Availability

Under the Research Health Act, the Government of Zambia does not allow public access to data collected in Zambia. All investigators interested in the data are required to submit a written request to the Ministry of Health. Contact Dr. Catherine Sutcliffe (csutcli1@jhu.edu) to coordinate the request.

## Abbreviations

%SpO_2_: Peripheral capillary oxygen saturation
adjPR: Age-adjusted prevalence ratio
adjRR: Age-adjusted risk ratio
ARI: Acute respiratory infection
BMI: Body mass index
CI: Confidence interval
ILI: Influenza-like illness
N/A: Not available
OPD: Outpatient department
PR: Prevalence ratio
RR: Risk ratio
RSV: Respiratory syncytial virus
RTI: Respiratory tract infection
