# Supplementary material for: Facility-based surveillance for influenza and respiratory syncytial virus in rural Zambia
Source: BMC Infect Dis. 2021 Sep 21;21:986. doi: 10.1186/s12879-021-06677-5 (PMC8453466; doi:10.1186/s12879-021-06677-5)
Supplement: Supplementary file 1 — Additional file 1: Sampling scheme for participants with influenza-like illness recruited from the outpatient department at Macha Hospital [file 12879_2021_6677_MOESM1_ESM.docx]

**Additional File 1.** **Sampling scheme for participants with influenza-like illness recruited from the outpatient department at Macha Hospital.**

| Age-group | Number sampled per week* | Number sampled per e*xpanded sampling* week ^‡^ |
| --- | --- | --- |
| 0 months – 11 months | 2 | 4 |
| 12 months – 59 months | 2 | 4 |
| 5 years – 15 years | 1 | 2 |
| 16 years – 50 years | 1 | 2 |
| > 50 years | 1 | 2 |

* The distribution of participants by age group was determined based on a review of the literature at the time the surveillance platform was designed to maximize detection of influenza cases. Patients with ILI within each age group were enrolled as they were identified until the target was met.

^‡^ Expanded week refers to the two weeks following the detection of an influenza-positive specimen.
