# Supplementary material for: Facility-based surveillance for influenza and respiratory syncytial virus in rural Zambia
Source: BMC Infect Dis. 2021 Sep 21;21:986. doi: 10.1186/s12879-021-06677-5 (PMC8453466; doi:10.1186/s12879-021-06677-5)
Supplement: Supplementary file 2 — Additional file 2: Symptomatology and clinical presentation of influenza virus and RSV infections by age [file 12879_2021_6677_MOESM2_ESM.docx]

**Additional File 2: Symptomatology and clinical presentation of influenza virus and RSV infections by age.**

| **Age <5 years** | | | | | | | | |
| --- | --- | --- | --- | --- | --- | --- | --- | --- |
|  | | **Total***  **n=398** | **Influenza A-infected**  **n=50** | | **Influenza B-infected**  **n=20** | | **RSV-infected**  **n=65** | |
|  |  |  | **Prevalence*** | **Univariable Prevalence Ratio (95% CI)** | **Prevalence*** | **Univariable Prevalence Ratio (95% CI)** | **Prevalence*** | **Univariable Prevalence Ratio (95% CI)** |
| **Duration of ILI symptoms at presentation, days** | median (IQR)^‡^ |  | 3 (3-5) | 1.00  (0.84-1.19) | 3 (2-3.5) | 0.89  (0.65-1.21) | 3 (3-4) | 1.06  (0.94-1.20) |
|  | 0-2 days | 102 | 12 (12%) | REF | 7 (7%) | REF | 11 (11%) | REF |
|  | 3-5 days | 249 | 34 (14%) | 1.16  (0.63-2.15) | 10 (4%) | 0.59  (0.23-1.50) | 47 (19%) | 1.75  (0.95-3.24) |
|  | 6+ days | 48 | 4 (8%) | 0.71  (0.24-2.08) | 3 (6%) | 0.91  (0.25-3.37) | 7 (15%) | 1.35  (0.56-3.27) |
| **Objective fever (n,%)** | No | 331 | 34 (10%) | REF | 14 (4%) | REF | 58 (18%) | REF |
|  | Yes | 68 | 16 (24%) | **2.29**  **(1.34-3.91)** | 6 (9%) | 2.09  (0.83-5.24) | 7 (10%) | 0.59  (0.28-1.23) |
| **Subjective fever (n,%)** | No | 3 | 0 (0%) | REF | 0 (0%) | REF | 1 (33%) | REF |
|  | Yes | 395 | 50 (13%) | N/A | 20 (5%) | N/A | 64 (16%) | 0.49  (0.10-2.45) |
| **Cough (n,%)** | No | 1 | 0 (0%) | REF | 0 (0%) | REF | 0 (0%) | REF |
|  | Yes | 396 | 50 (13%) | N/A | 19 (5%) | N/A | 65 (16%) | N/A |
| **Sore throat (n,%)** | No | 139 | 15 (11%) | REF | 3 (2%) | REF | 30 (22%) | REF |
|  | Yes | 6 | 0 (0%) | N/A | 0 (0%) | N/A | 3 (50%) | 2.32  (0.98-5.48) |
| **Headache (n,%)** | No | 88 | 8 (9%) | REF | 1 (1%) | REF | 24 (27%) | REF |
|  | Yes | 27 | 3 (11%) | 1.22  (0.35-4.29) | 3 (11%) | **9.78**  **(1.06-90.18)** | 4 (15%) | 0.54  (0.21-1.43) |
| **Tachypnea (n,%)** | No | 382 | 50 (13%) | REF | 20 (5%) | REF | 63 (17%) | REF |
|  | Yes | 17 | 0 (0%) | N/A | 0 (0%) | N/A | 2 (12%) | 0.71  (0.19-2.67) |
| **Hypoxemia^†^  (n,%)** | No | 226 | 36 (16%) | REF | 15 (7%) | REF | 21 (9%) | REF |
|  | Yes | 59 | 10 (17%) | 1.06  (0.56-2.02) | 2 (3%) | 0.51  (0.12-2.17) | 17 (29%) | **3.10**  **(1.75-5.49)** |
| **Diarrhea (n,%)** | No | 283 | 37 (13%) | REF | 16 (6%) | REF | 54 (19%) | REF |
|  | Yes | 115 | 12 (10%) | 0.80  (0.43-1.47) | 4 (3%) | 0.62  (0.21-1.80) | 11 (10%) | **0.50**  **(0.27-0.92)** |
| **Age ≥ 5 years** | | | | | | | | |
|  | | **Total***  **n=273** | **Influenza A-infected***  **n=35** | | **Influenza B-infected***  **n=17** | | **RSV-infected***  **n=12** | |
|  |  |  | ***Prevalence*** | ***Univariable*  Prevalence *Ratio (95% CI)*** | ***Prevalence*** | ***Univariable*  Prevalence *Ratio (95% CI)*** | ***Prevalence*** | ***Univariable*  Prevalence *Ratio (95% CI)*** |
| **Duration of ILI symptoms at presentation, days** | median (IQR) ^‡^ | 4 | 3 (3-4.5) | 0.94  (0.77-1.15) | 4 (3-4) | 0.90  (0.65-1.24) | 4 (4-5) | 1.01  (0.79-1.30) |
|  | 0-2 days | 32 | 3 (9%) | REF | 2 (6%) | REF | 1 (3%) | REF |
|  | 3-5 days | 191 | 28 (15%) | 1.56  (0.50-4.84) | 12 (6%) | 1.01  (0.24-4.28) | 9 (5%) | 1.51  (0.20-11.50) |
|  | 6+ days | 48 | 5 (10%) | 1.11  (0.29-4.33) | 2 (4%) | 0.67  (0.10-4.49) | 1 (2%) | 0.67  (0.04-10.28) |
| **Objective fever (n,%)** | No | 231 | 26 (11%) | REF | 7 (3%) | REF | 9 (4%) | REF |
|  | Yes | 39 | 10 (25%) | **2.28**  **(1.19-4.34)** | 9 (22%) | **7.62**  **(3.01-19.26)** | 2 (5%) | 1.32  (0.30-5.86) |
| **Subjective (n,%)** | No | 1 | 1 (100%) | REF | 0 (0%) | REF | 0 (0%) | REF |
|  | Yes | 271 | 35 (13%) | N/A | 16 (6%) | N/A | 11 (4%) | N/A |
| **Cough (n,%)** | No | 10 | 0 (0%) | REF | 0 (0%) | REF | 0 (0%) | REF |
|  | Yes | 261 | 36 (14%) | N/A | 16 (6%) | N/A | 11 (4%) | N/A |
| **Sore throat (n,%)** | No | 188 | 30 (16%) | REF | 11 (6%) | REF | 6 (3%) | REF |
|  | Yes | 71 | 5 (7%) | 0.44  (0.18-1.09) | 5 (7%) | 1.20  (0.43-3.34) | 2 (3%) | 0.88  (0.18-4.27) |
| **Headache (n,%)** | No | 68 | 12 (18%) | REF | 4 (6%) | REF | 4 (6%) | REF |
|  | Yes | 196 | 23 (12%) | 0.66  (0.35-1.26) | 12 (6%) | 1.04  (0.35-3.12) | 5 (3%) | 0.43  (0.12-1.57) |
| **Tachypnea (n,%)** | No | 112 | 7 (6%) | REF | 3 (3%) | REF | 3 (3%) | REF |
|  | Yes | 160 | 29 (18%) | **2.90**  **(1.32-6.39)** | 13 (8%) | 3.03  (0.88-10.40) | 8 (5%) | 1.87  (0.51-6.88) |
| **Hypoxemia^†^ (n,%)** | No | 179 | 27 (15%) | REF | 14 (8%) | REF | 7 (4%) | REF |
|  | Yes | 22 | 3 (14%) | 0.90  (0.30-2.74) | 2 (9%) | 1.16  (0.28-4.78) | 1 (5%) | 1.16  (0.15-9.01) |
| **Diarrhea (n,%)** | No | 240 | 30 (12%) | REF | 12 (5%) | REF | 11 (5%) | REF |
|  | Yes | 32 | 6 (19%) | 1.50  (0.68-3.32) | 4 (12%) | 2.50  (0.86-7.29) | 0 (0%) | N/A |

^*^Percentages represent row totals (i.e. the total number with a given attribute within each infection group divided by the total number with a given attribute).

^‡^ Prevalence ratio represents the change per day in duration of symptoms

^†^ Hypoxemia: SpO_2_<93% at the time of enrollment.

CI: Confidence interval; ILI: influenza-like illness; IQR: Interquartile range; N/A: Not available; REF: Reference group; Bold = p<0.05 from log-binomial regression
