# Supplementary material for: Facility-based surveillance for influenza and respiratory syncytial virus in rural Zambia
Source: BMC Infect Dis. 2021 Sep 21;21:986. doi: 10.1186/s12879-021-06677-5 (PMC8453466; doi:10.1186/s12879-021-06677-5)
Supplement: Supplementary file 3 — Additional file 3: Age-specific seasonal trends over time in A) influenza-like illness, B) influenza A virus, C) influenza B virus, and D) respiratory syncytial virus prevalence among outpatients in Macha, Zambia, December 2018 to December 2019. [file 12879_2021_6677_MOESM3_ESM.docx]

**Additional File 3. Age-specific trends in A) influenza-like illness, B) influenza A virus, C) influenza B virus, and D) respiratory syncytial virus prevalence among outpatients in Macha, Zambia, December 2018 to December 2019.**

ILI: influenza-like illness; RSV: respiratory syncytial virus
