# Supplementary material for: Facility-based surveillance for influenza and respiratory syncytial virus in rural Zambia
Source: BMC Infect Dis. 2021 Sep 21;21:986. doi: 10.1186/s12879-021-06677-5 (PMC8453466; doi:10.1186/s12879-021-06677-5)
Supplement: Supplementary file 4 — Additional file 4: Age-distribution of all outpatients, outpatients with influenza-like illness, and outpatient study participants [file 12879_2021_6677_MOESM4_ESM.docx]

**Additional File 4.** **Age-distribution of all outpatients, outpatients with influenza-like illness, and outpatient study participants.**


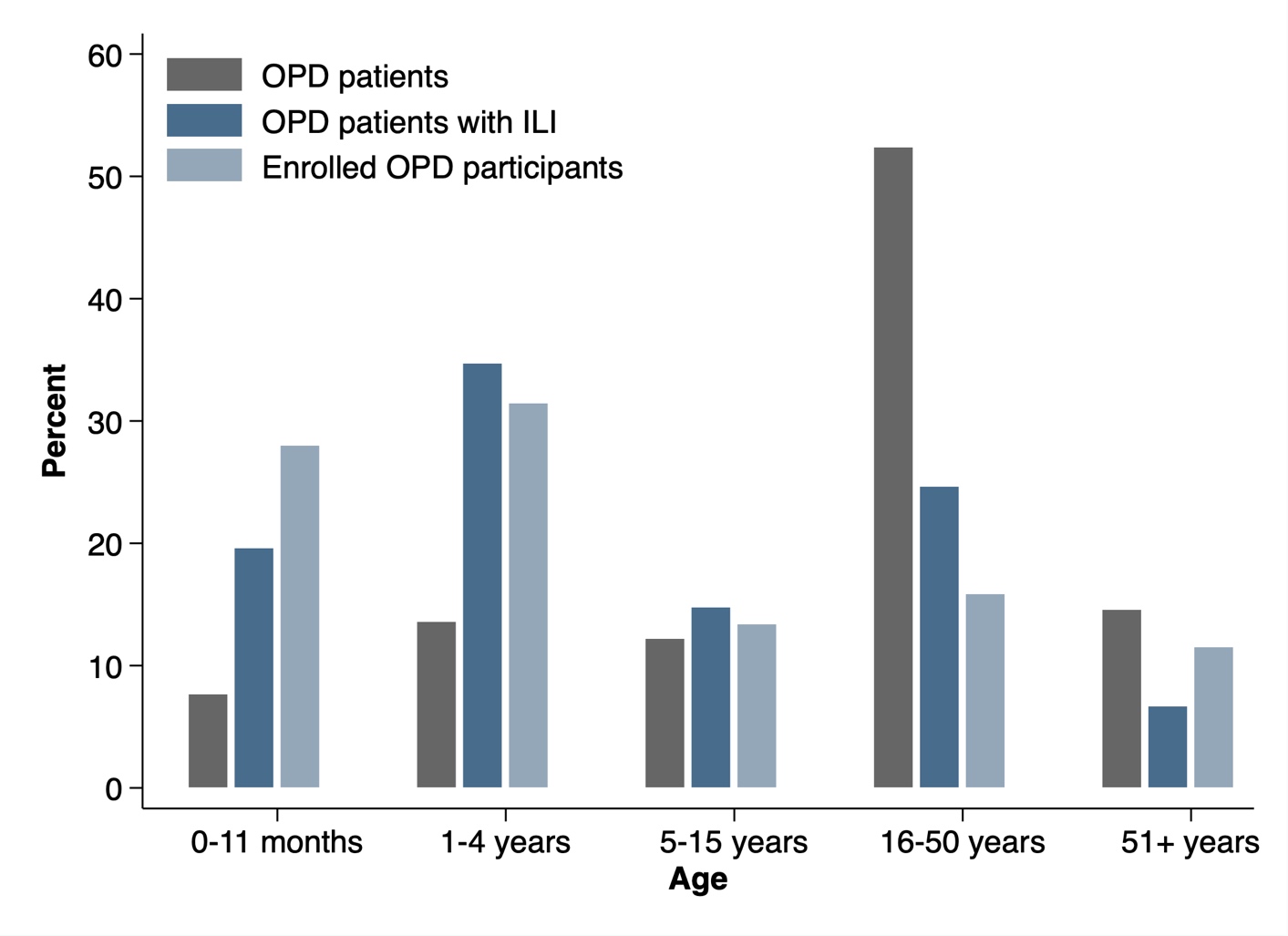


OPD: outpatient department; ILI: influenza-like illness. Denominator for each proportion is all OPD patients (gray bar); OPD patients with ILI (dark blue bar); and OPD participants (gray-blue bar).
