# Supplementary material for: Facility-based surveillance for influenza and respiratory syncytial virus in rural Zambia
Source: BMC Infect Dis. 2021 Sep 21;21:986. doi: 10.1186/s12879-021-06677-5 (PMC8453466; doi:10.1186/s12879-021-06677-5)
Supplement: Supplementary file 5 — Additional file 5: Age-distribution of all inpatients, inpatients with acute respiratory illness, and inpatient study participants [file 12879_2021_6677_MOESM5_ESM.docx]

**Additional File 5. Age-distribution of all inpatients, inpatients with acute respiratory illness, and inpatient study participants.**


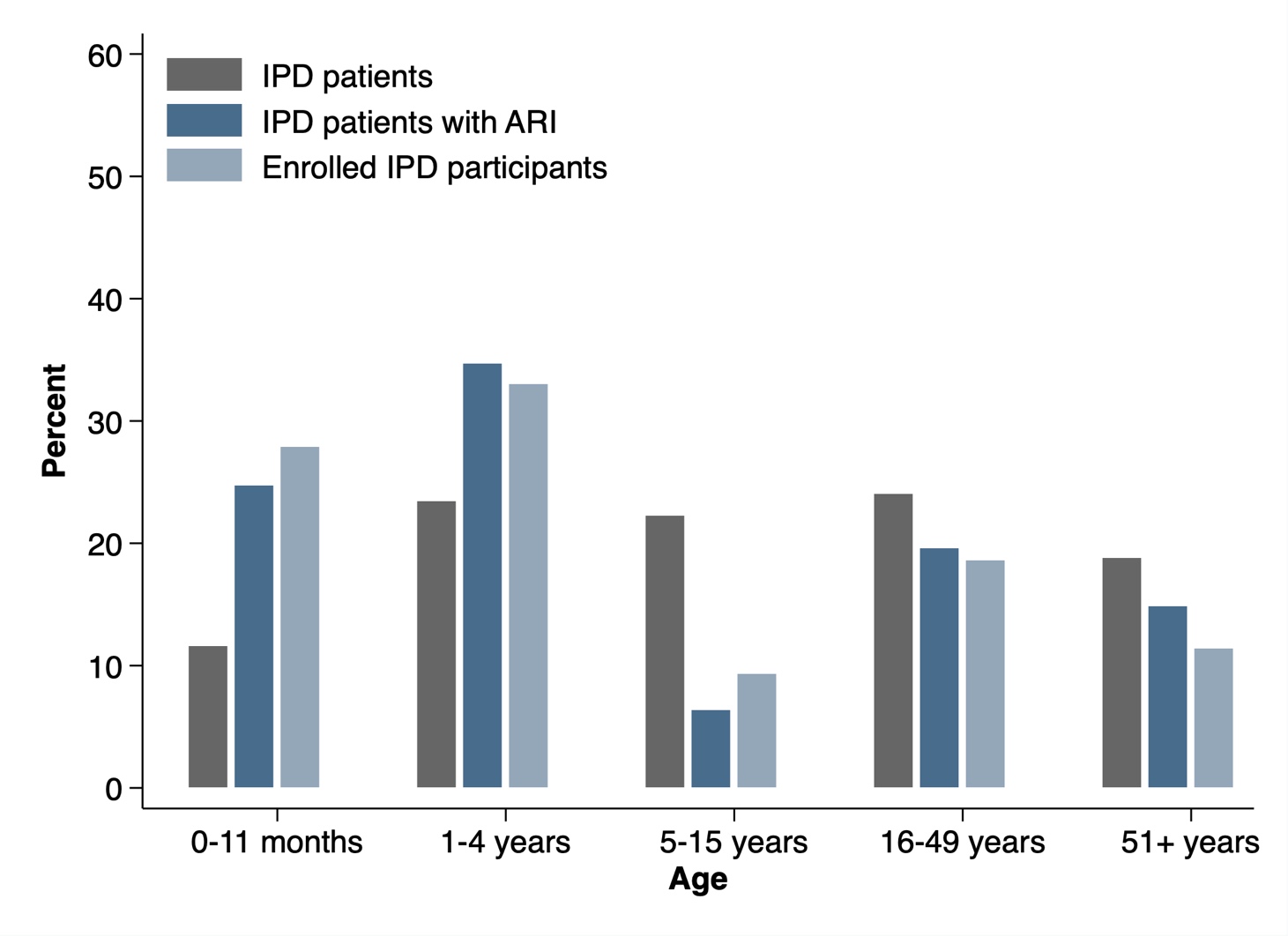


IPD: inpatient department; ARI: acute respiratory illness. Denominator for each proportion is all newly admitted patients (gray bar); newly admitted patients with ARI (dark blue bar); and IPD participants (gray-blue bar).
