# Supplementary material for: Facility-based surveillance for influenza and respiratory syncytial virus in rural Zambia
Source: BMC Infect Dis. 2021 Sep 21;21:986. doi: 10.1186/s12879-021-06677-5 (PMC8453466; doi:10.1186/s12879-021-06677-5)

**Additional File 6**.  **Temperature, precipitation, and viral infection in Macha Zambia, December 2018 to December 2019.**


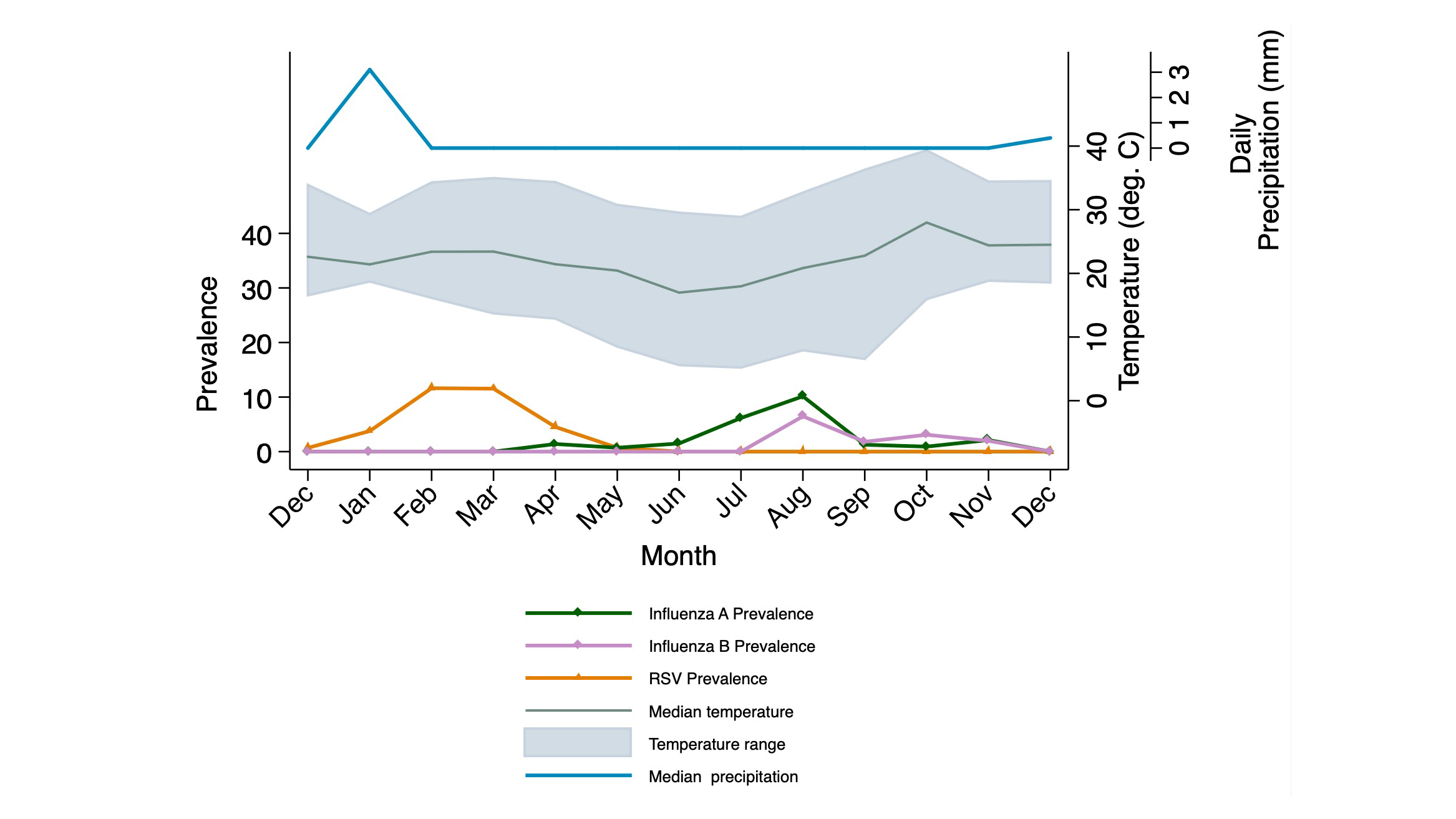

Supplement: Supplementary file 6 — Additional file 6: Temperature, precipitation, and viral infection in Macha Zambia, December 2018 to December 2019. [file 12879_2021_6677_MOESM6_ESM.docx]
